# Magnetic resonance imaging of fetal vascular malformations

**DOI:** 10.1007/s00247-024-05983-9

**Published:** 2024-07-09

**Authors:** Willemijn M. Klein, Georgia Papaioannou

**Affiliations:** 1 Department of Medical Imaging, Amalia Children’s Hospital, Radboud University Medical Center, Nijmegen, the Netherlands; 2 Expertise Center for Hemangiomas and Congenital Vascular Malformations Nijmegen (Hecovan), Radboud University Medical Center, Nijmegen, the Netherlands; 3 Paediatric Radiology Department, Mitera Maternity and Children’s Hospital, Athens, Greece

**Keywords:** Arteriovenous malformation, Hemangioma, Lymphatic abnormalities, Neoplasm, Prenatal diagnosis, Radiology, Vascular malformations

## Abstract

Vascular anomalies develop during fetal life and can be detected on prenatal ultrasonography and fetal magnetic resonance imaging. Diagnosis of lymphatic, venous, and arteriovenous malformations, as well as congenital hemangiomas and other congenital vascular tumors, may be challenging. The benign vascular anomalies may be difficult to differentiate from malignancies with a similar appearance. In this manuscript, we present a succinct overview of the congenital vascular anomalies that may present in fetal or neonatal life.

## Introduction

Congenital vascular abnormalities are rare and may be difficult to recognize and accurately diagnose, especially during the fetal stage of life. The Society for the Study of Vascular Anomalies (ISSVA) has classified the congenital vascular abnormalities, including vascular malformations (which are developmental abnormalities of vessels) and vascular tumors (which are proliferative endothelial lesions) [1]. Many of the vascular anomalies develop during fetal life and these can be detected during prenatal ultrasonography (US). In addition to US, fetal magnetic resonance imaging (MRI) can be used to narrow the differential diagnosis, evaluate the localization and extension of vascular anomalies, and prepare the medical support during and after birth. This article provides an overview of fetal MRI presenting various types of vascular malformations, including typical appearances as well as potential mimickers.

## Magnetic resonance technique

Imaging a fetus can be challenging. Sedation of the mother is usually not required. The small and active fetus necessitates specific imaging techniques to visualize finer details. The high spatial and temporal resolution of ultrasonography are sufficiently diagnostic in most cases of congenital abnormalities. MRI is indicated in cases where the fetal position is too deep within the abdomen or when an abnormality lies beneath the ossified bones which makes it difficult to visualize with US. Moreover, the various MR sequences enable better tissue differentiation [2].

The cystic and vascular anatomy of most low-flow vascular malformations is depicted with high-resolution, single-shot T2-weighted images, in two or preferably three planes. High-flow artifacts can be depicted on T2-weighted images, whereas ultrafast three-dimensional (D) real-time cine sequences are useful in providing details of the cardiovascular anatomy. T1-weighted images are applied to demonstrate protein-rich areas, which can be static blood, thrombosis, hemorrhage, or meconium. Finally, the T2* sequence is useful in detecting hemosiderin deposits. 3 tesla (T) MRI generally results in higher quality images compared to 1.5T. Diffusion-weighted imaging (DWI) is technically challenging in fetuses, and its value in fetal vascular malformations is, as yet, not established. Nevertheless, it may be helpful in the differential diagnosis.

## Classification of vascular anomalies

Vascular anomalies are classified as either *developmental malformations*, which are vessels with abnormal morphogenesis during the embryological and fetal period, or *vascular tumors*, which have abnormal cell proliferation [1]. It is helpful for diagnostic classification to further divide abnormalities into low-flow and high-flow lesions. Low-flow developmental malformations are venous or lymphatic malformations or a combination of both (veno-lymphatic malformation), consisting of cysts or varicose and ectatic venous or lymphatic vessels. High-flow developmental malformations are arteriovenous malformations, consisting of arteries directly connected to veins, both having triphasic flow. Capillary malformations are located superficially in the skin and are usually not visualized with (fetal) imaging; however, these can exist in combination with venous or arteriovenous malformations. Vascular tumors consist of solid tissue with a prominent component of high-flow vessels and abnormal proliferating endothelial cells. These are either congenital (develop in the fetus) or infantile (develop in the neonate) hemangiomas, which are both benign, or tumors with more aggressive behaviors, such as kaposiform hemangioendothelioma (Table 1). Other soft tissue tumors that may have a hypervascular appearance but without a vascular origin, for example, sarcomas, are not classified as vascular tumors.

**Table 1:** Vascular anomalies that can develop during the fetal period and present on fetal imaging or in the perinatal period

	Flow	Appearance on ultrasound and magnetic resonancei imaging	Syndrome or complex	Mimicker	Genotype (typical)
Vascular malformation					
Lymphatic malformation	Low	Macrocystic, microcystic, tubular; internal hemorrhage with fluid-fluid levels and/or T2 mixed signal	Complex lymphatic anomaly	(Cystic) teratoma, rhabdomyosarcoma, extrapulmonary sequestration	*PIK3CA*
Venous malformation	Low	Ectatic vessels or lobulated mass, high on T2, solitary or multifocal, no flow void, fluid-fluid levels, thrombosis (high on T1) and calcified phleboliths (no signal)	*PTEN*-related overgrowth syndrome, Klippel-Trenaunay	Low-flow mass/tumor, nearly involuted hemangioma	*PIK3CA*, *TEK*, *PTEN*
Capillary malformation	Low	Superficial port wine stain, not visible on imaging; may have underlying arteriovenous or venous malformation	Sturge-Weber		*GNAQ*
Arteriovenous malformation	High	No pathological solid tissue. A high-flow vascular nidus with prominent tortuous afferent and efferent vessels, all with high-flow voids		Congenital hemangioma, hypervascular tumors	*MAP2K1*, *RASA1*
Vascular tumor					
Congenital hemangioma	High	Solid hypervascular lesion, growth until perinatal period, present at birth, involution either rapid, partial or non-involuting		Arteriovenous malformation, kaposiform hemangioendothelioma, infantile sarcoma, rhabdomyosarcoma, (solid) teratoma	*GNAC*, *GNA11*; *GLUT1* negative
Infantile hemangioma	High	Solid hypervascular lesion (low echogenicity on US, high signal on T2), appearance and proliferation in the first months; involution in the first years	LUMBAR, PELVIS/PHACES	Sarcoma, rhabdomyosarcoma, kaposiform hemangioendothelioma, venous malformation	*GLUT1*
Kaposiform hemangioendothelioma	High	Mass growing diffusely through fascia, locally aggressive, diffusely enhancing; may have Kasabach-Merritt phenomenon		Arteriovenous malformation, congenital hemangioma, infantile sarcoma, rhabdomyosarcoma, (solid) teratoma	*GNA14*

*LUMBAR* lower body hemangioma and other cutaneous defects; urogenital anomalies/ulceration; myelopathy; bony deformities; anorectal malformations/arterial anomalies; renal anomalies, *PELVIS* perineal hemangioma, external genitalia malformations, lipomyelomeningocele, vesicorenal abnormalities, imperforate anus, skin tag, *PHACES* posterior fossa brain malformations, hemangioma (facial), arterial anomalies, cardiac anomalies/coarctation of the aorta, eye abnormalities/endocrine abnormalities, sternal cleft/supraumbilical raphe

## Low-flow vascular malformations

### Lymphatic malformations

**Fig. 1:**
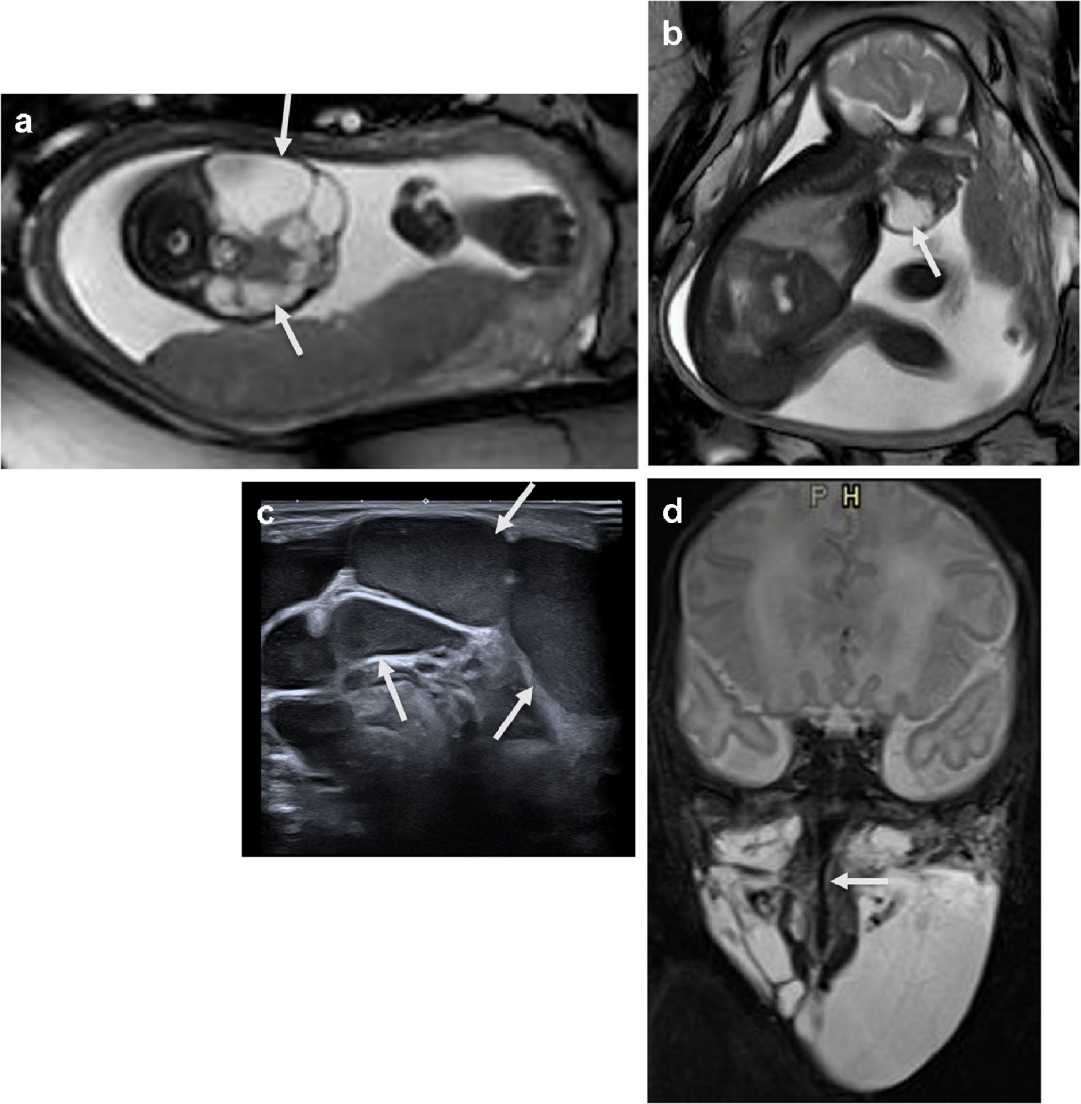
A female fetus of 31 weeks gestational age with a lymphatic malformation adjacent to the pharynx and trachea that required perinatal intervention to secure the airway. **a**, **b** Axial (**a**) and sagittal (**b**) T2-weighted magnetic resonance images demonstrate the macrocystic lymphatic malformation (*arrows*) in the submandibular region extending to the pharynx and trachea, without compression of these structures. A vaginal birth was anticipated and indeed the neonate presented no signs of airway obstruction after birth. **c** Ultrasound of the lymphatic malformation in the oblique plane, performed directly after term birth, demonstrates the dense fluid in several cysts, which could be hemorrhagic (*arrows*). **d** Postnatal coronal magnetic resonance image at day 2 shows slight compression of the trachea (*arrow*) caused by the surrounding lymphatic cysts

Simple lymphatic malformations are typically located along the course of the central lymphatic vessels, which is alongside the aortoiliac vessels in the retroperitoneum and mediastinum, and at the lymphatic outflow of the subclavian-jugular angles on the left and right side. The mesentery, which has many lymphatic vessels, is also a typical location for lymphatic malformations. Lymph vessels are found in various locations in the body and extremities and therefore lymphatic malformations can be found in almost any anatomical site. On fetal US and MRI, a lymphatic malformation presents as a multi-cystic mass, without a solid component or calcification and with very low or absent flow in the walls of the cysts. The cystic fluid may differ in signal per cyst, due to internal hemorrhage. A cystic lymphatic malformation may swell and compress the surrounding tissues, but it does not present as an invasive growth; expansion is caused by hemorrhaging within the cysts. If the lymphatic malformation is adjacent to the pharynx and the trachea, it may compress these airways and be life-threatening; therefore, an ex-utero intrapartum treatment (EXIT) may be considered in order to secure the airway (Fig. 1) [3]. In any location, the mass effect in cases of hemorrhaging and infection may cause an obstruction to adjacent structures, or be painful.

**Fig. 2:**
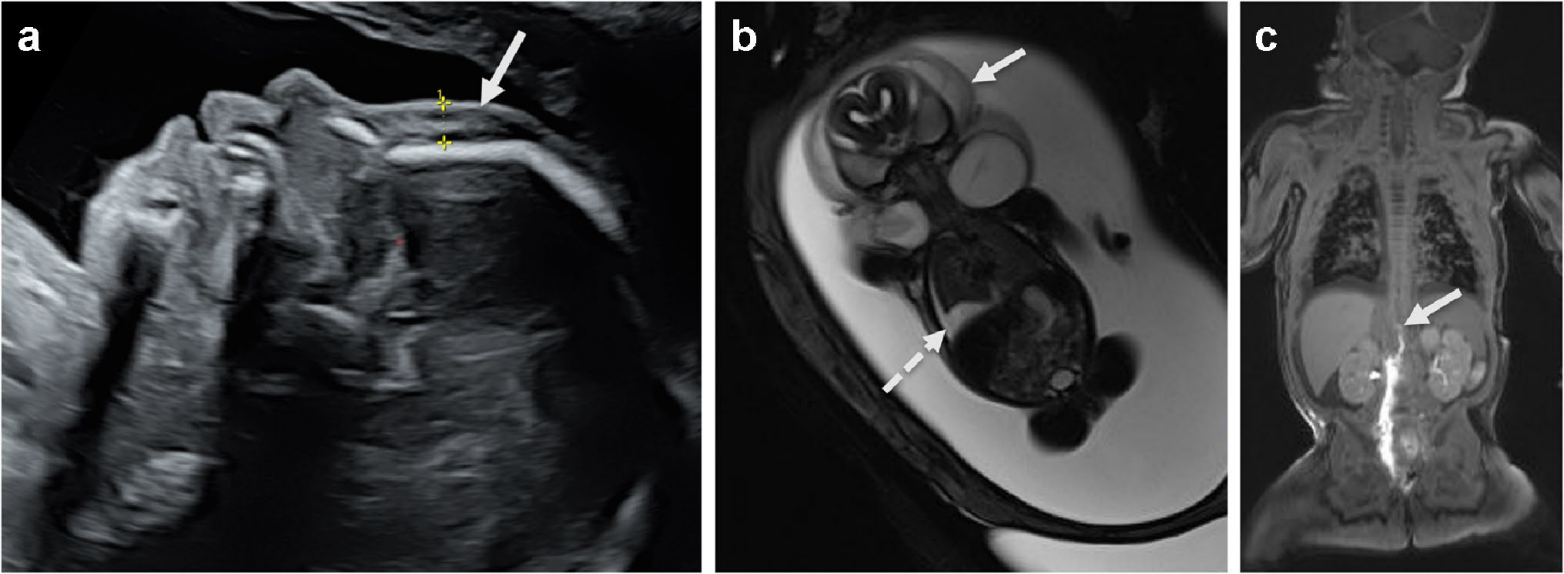
Complex lymphatic anomaly cases. **a**,** b** Prenatal sagittal ultrasound (**a**) and coronal magnetic resonance (**b**) images of a male fetus at 23 weeks gestational age. **a** Demonstrates extensive subcutaneous edema (*arrow*) with the calipers measuring 8 mm thickness. **b** Demonstrates extensive subcutaneous edema (*arrow*) and pleural fluid (*broken* *arrow*), consistent with fetal hydrops. A *SOS1* mutation was identified, consistent with Noonan syndrome. **c** Coronal T1-weighted magnetic resonance lymphangiography image after intranodal contrast injection, in a similar case of fetal hydrops and Noonan syndrome (female neonate, 4 weeks). There is contrast enhancement in the right groin and retroperitoneal lymphatic vessels. The enhancement ends (*arrow*) below the thoracic duct suggesting aplasia

In addition to simple lymphatic malformations, complex lymphatic anomalies consisting of malformed lymphatic vessels are also recognized. These may co-exist with lymphatic cysts located in the liver, spleen, or bones, categorized as generalized lymphatic anomalies. Complex lymphatic anomalies can be tumorlike and growing as in kaposiform lymphatic anomalies, or have lymphatic flow disturbances due to aplasia or obstruction of the thoracic duct, labeled as central conducting lymphatic anomalies. These three types of complex lymphatic anomalies may overlap. Complex lymphatic anomalies, especially central conducting lymphatic anomaly, are one of the causes of fetal hydrops (Fig. 2) and congenital hydro-/chylothorax, often in Noonan-like syndromes, and need to be considered in the differential diagnosis. The recognition of the actual ectatic and aplastic lymphatic vessels on fetal T2-weighted MR images is challenging. The diagnosis complex lymphatic anomaly can be confirmed with postnatal MR lymphangiography with intranodal contrast agent injection to enhance the lymphatic vessels (Fig. 2) [4].

Congenital multi-cystic masses that may look similar to lymphatic anomalies are, for instance, sacrococcygeal or faciocervical teratomas, which often present with areas of internal hemorrhage, typically contain fat and calcifications. Remodeling and scalloping of the adjacent bone may occur with teratomas, but not with lymphatic malformations. Rhabdomyosarcoma in the head and neck may resemble a lymphatic anomaly or as a painless mass with cysts; however, congenital rhabdomyosarcoma is often aggressive with rapid growth of solid components and metastases at presentation [5]. Lesions that raise diagnostic uncertainties may need further (postnatal) imaging and possibly a biopsy.

### Venous malformations

**Fig. 3:**
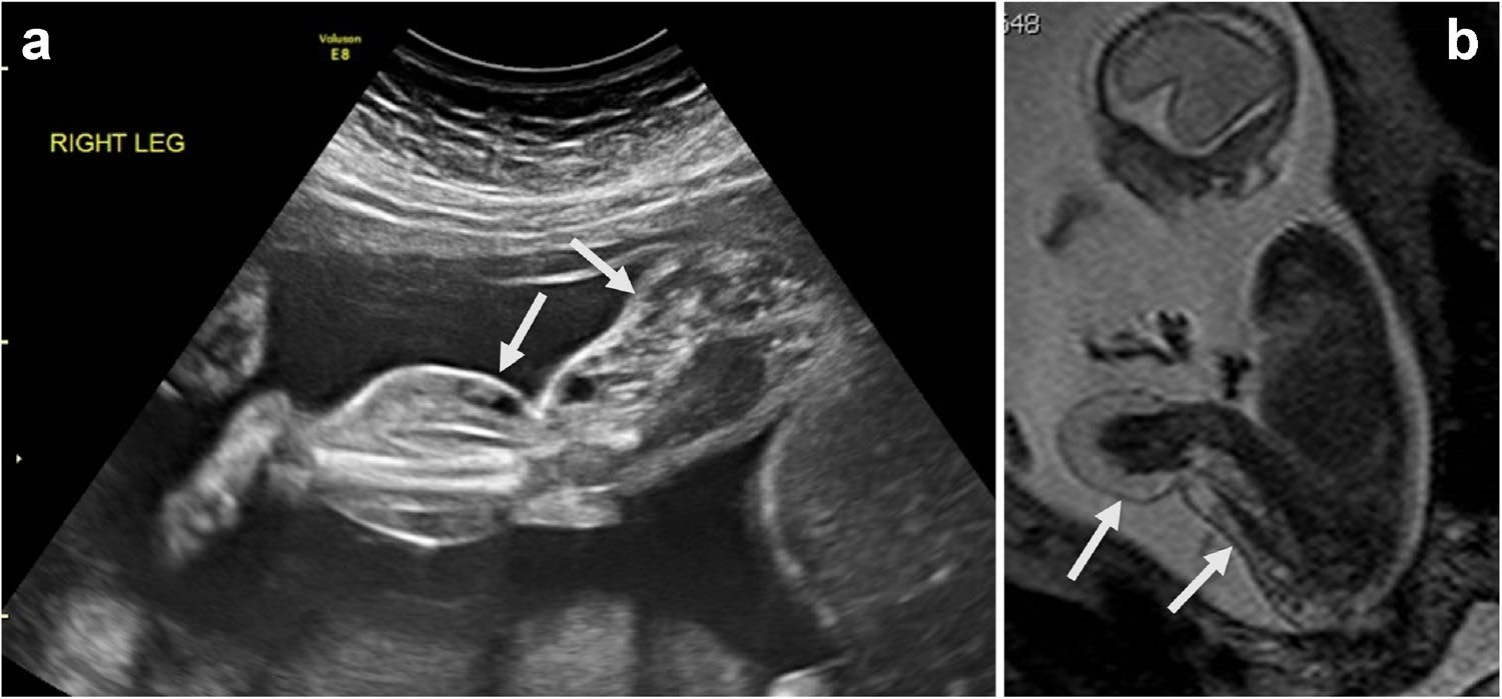
Venous malformation with overgrowth in a female fetus at 23-week gestational age. **a**, **b** Prenatal oblique ultrasound (**a**) and sagittal T2-weighted fetal magnetic resonance (**b**) images show multiple anechoic cystic and low-flow vascular structures and thickening of the subcutaneous fat (*arrows*), suggestive of a venous malformation with soft-tissue overgrowth. This fetus had genetically confirmed *PIK3CA* related overgrowth syndrome

Venous malformations can occur at any location but are often found in the extremities, in the muscles and subcutaneous spaces. A venous malformation consists of ectatic or near-cystic venous vessels with very slow flow. A simple, solitary venous malformation is not usually found in fetuses and neonates unless substantial in size. It generally presents after birth with discoloration of the skin, or when hindering motion due to an intramuscular location and rarely when painful due to internal thrombosis, which usually occurs (or is noticed) later in life. Focal lesions within the liver, spleen, and bones, typically found in adults, may be misnamed as hemangiomas, whereas these are actually low-flow venous malformations. On MRI, a venous malformation has high T2 signal and low or intermediate T1 signal. Thrombi within the malformation may appear as linear structures with high T1 signal and calcified phleboliths as ovoid structures with low signal on T1- and T2-weighted images. A venous malformation may be well demarcated or extend through facias, or may appear as extensive varicose veins. It may be accompanied with overgrowth in Klippel-Trenaunay syndrome and *PIK3CA*-related overgrowth syndromes (PROS) (Fig. 3).

Several lesions may mimic a venous malformation in the fetus and neonate. A fibro-adipose vascular anomaly is a venous malformation combined with fibrofatty and muscular infiltration, typically in the gastrocnemius muscle. A soft tissue hamartoma, also called angiomatosis and associated with *PTEN* genotype, typically shows dilated draining veins, fibrous and adipose tissue, without respect to fascial planes, and often multifocal. A spindle cell hemangioma typically has dilated and thrombosed vessels with phleboliths and may look similar to a benign venous malformation or a low-grade sarcoma on histology. Although rare, there are malignant lesions that appear similar to venous malformations, especially congenital fibrosarcoma: a solid, firm mass that grows, with discoloration of an extremity, due to large internal hemorrhaging and ulceration, combined with thrombocytopenia and coagulopathies. It may resemble both congenital hemangiomas and venous malformations. Histological tissue examination and genotype are required to obtain a definite diagnosis [6].

## High-flow vascular anomalies

### Arteriovenous malformations

**Fig. 4:**
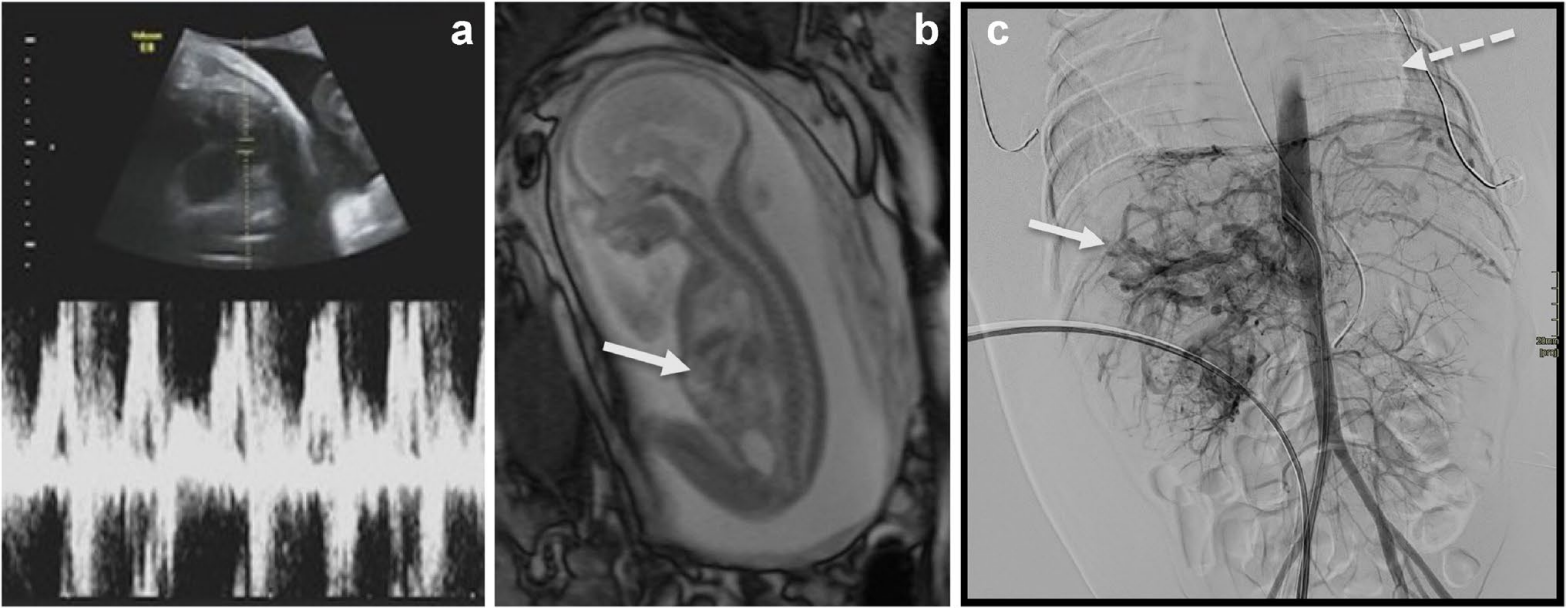
Arteriovenous malformation of the liver in a female fetus at 23 weeks gestational age. **a** Prenatal oblique ultrasound demonstrates high-flow signal in the liver. **b** Fetal sagittal T2-weighted magnetic resonance image demonstrates the high-flow signal voids in the liver (*arrow*), without soft-tissue mass, differentiating this lesion from congenital hemangioma. **c** Postnatal coronal angiographic image of this high-flow lesion (*arrow*) shows extensive shunting and cardiomegaly (*broken* *arrow*). The diagnosis of an arteriovenous malformation was confirmed at autopsy

**Fig. 5:**
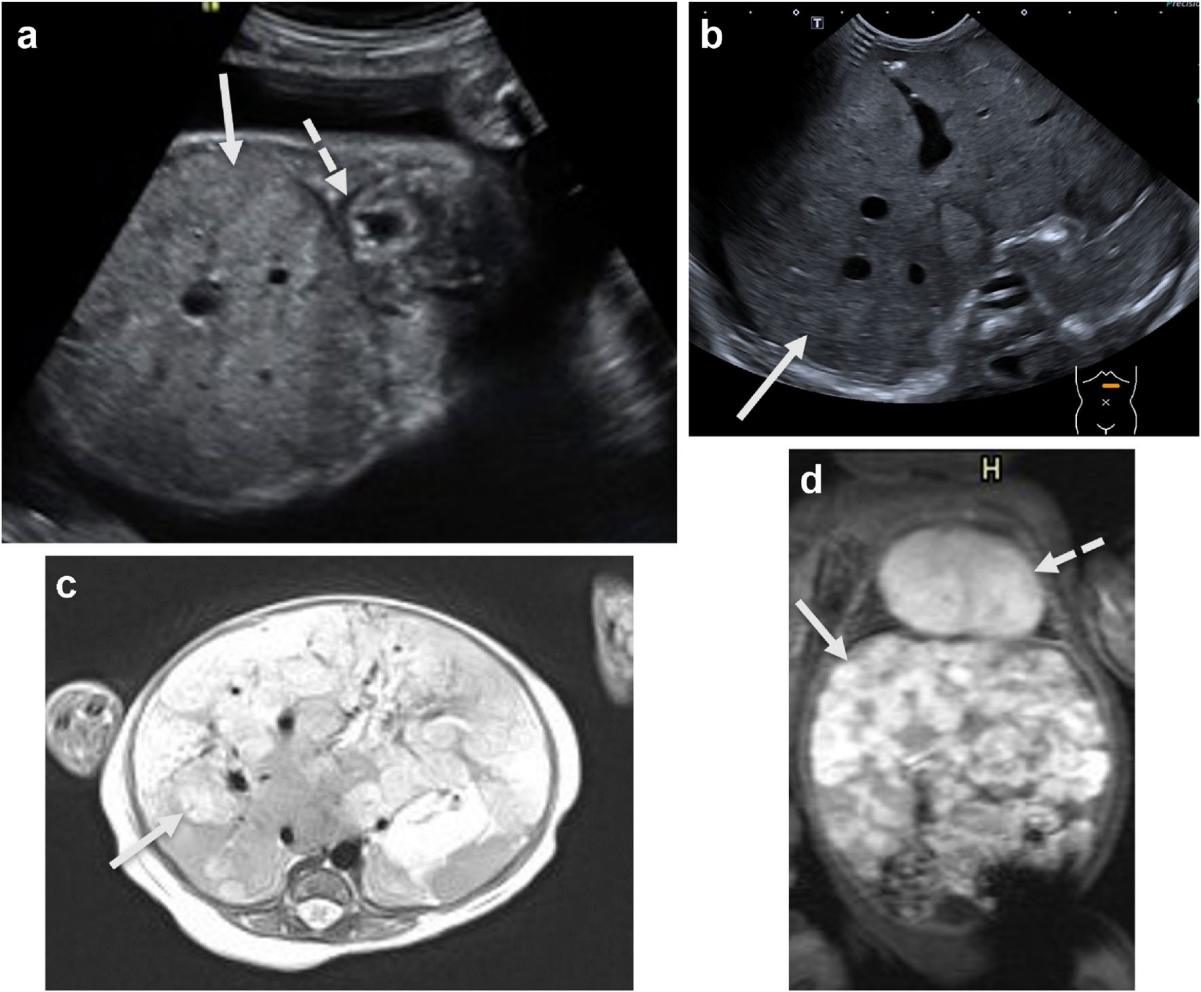
Congenital hemangiomas of the liver in a female fetus and neonate. **a** Prenatal coronal ultrasound at 30 weeks gestational age, demonstrates an enlarged inhomogeneous liver (*arrow*) and some pericardial fluid (*broken arrow*). Additionally, cardiomegaly, multiple skin lesions and some ascites were identified (not shown). The liver abnormalities were consistent with the diagnosis of hepatic hemangiomatosis. **b**–**d** Postnatal transverse ultrasound (**b**), axial T2-weighted magnetic resonance (**c**)  and coronal T1-weighted postcontrast magnetic resoance (**d**) images after delivery at 32 weeks gestational age, demonstrate multiple nodular liver lesions (*arrows*) with high vascularity, causing extensive shunting and cardiomegaly (*broken* *arrow*)

**Fig. 6:**
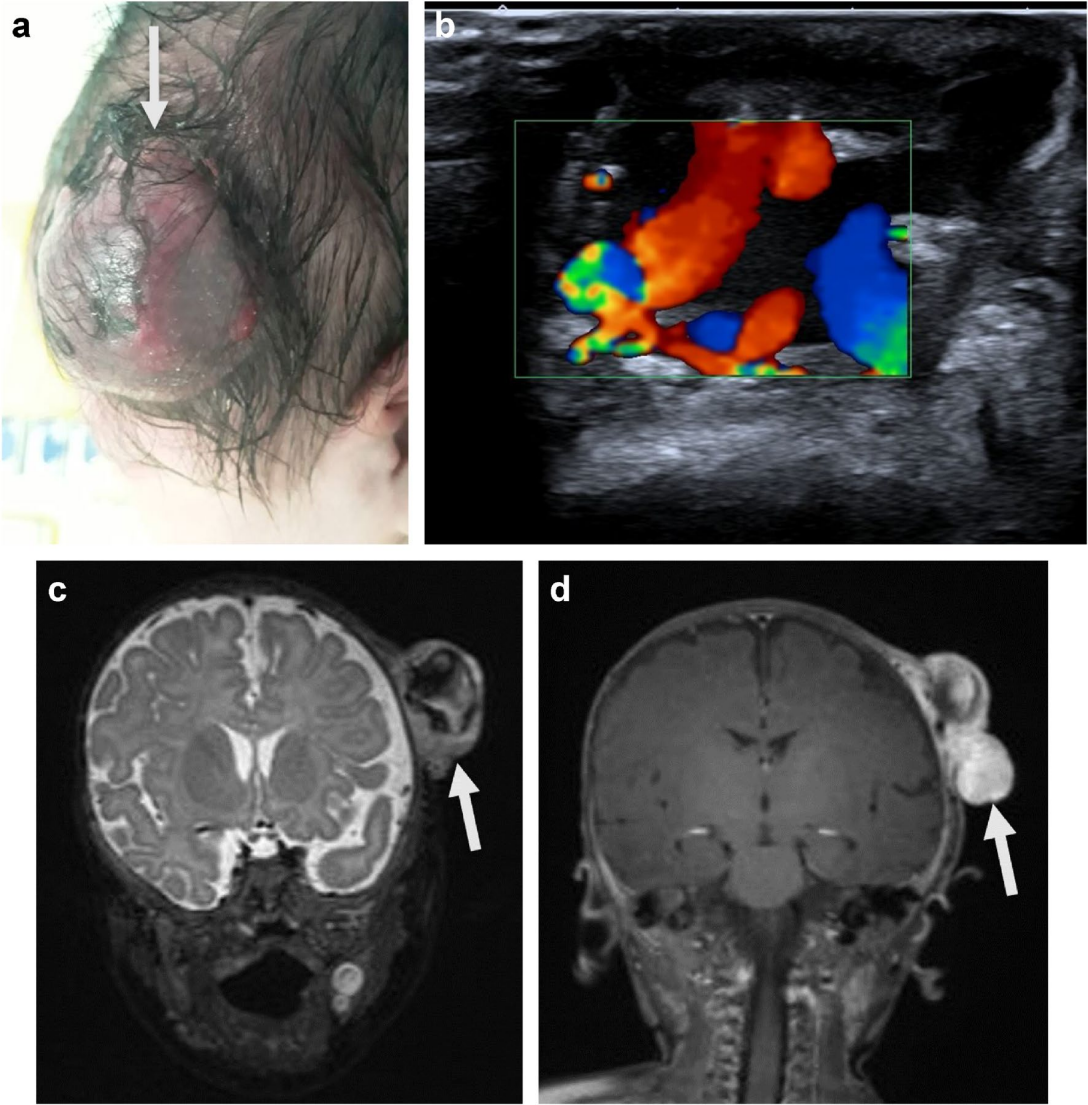
Congenital sarcoma in a boy at 4 months of age. **a** A solid purple lesion was noted in the scalp at birth (*arrow*). It was initially diagnosed as a congenital hemangioma. However, due to continuous growth in the first months of life, the diagnosis was reconsidered. **b** Oblique Doppler ultrasound image shows turbulent (multicolor) high flow within the mass. **c **Coronal T2-weighted magnetic resonance image shows inhomogeneous signal intensity within the lesion. **d **Postcontrast coronal T1-weighted magnetic resonance image shows enhancement of the lesion extending to the surrounding soft-tissues, which is not typical of a congenital hemangioma. Biopsy confirmed spindle cell sarcoma

An arteriovenous malformation consists of arteries and veins with abnormal direct connections, without an intervening capillary network; as a result, the high triphasic flow in the afferent arteries continues in the efferent veins. This tangle of abnormal vessels includes no pathologic solid tissue, although there may be some surrounding edema secondary to the abnormal circulation. An arteriovenous malformation can show angiogenesis and growth [7]. A large arteriovenous malformation can result in left-to-right shunting and cardiac overload, with an additional risk of (massive) bleeding, either of which may be life-threatening for the fetus and neonate. Therefore, in these cases, intensive perinatal care is required. These high-flow lesions, typically in the liver parenchyma, can be detected on fetal Doppler US [Fig. 4]. A fetal MR scan can be performed to distinguish arteriovenous malformations from other mimicking hypervascular lesions that do include solid components, usually congenital hemangioma (Fig. 5) or rarely a congenital sarcoma (Fig. 6). However, small arteriovenous malformations are usually not detected prenatally and will only come to the attention later in life, due to its growth, pain, and impairment.

### Vascular tumors

High-flow solid masses in the fetus may be congenital hemangiomas, which are benign, or more aggressive vascular tumors, such as kaposiform hemangioendothelioma (KHE).

#### Congenital hemangioma

Hemangiomas have a rapid proliferation phase and subsequently involute. *Congenital* hemangiomas have their growing phase during fetal life and can be detected with prenatal US and MRI. These congenital hemangiomas are subdivided as either rapid, partially, or non-involuting, which describes the pace of involution during postnatal life. Conversely, infantile hemangiomas do not appear during fetal life per definition and proliferate postnatally during the first year, after which they slowly involute over up to 10 years. Congenital and infantile hemangiomas have different genotypes, with the congenital type being *GLUT-1* negative and the infantile type being *GLUT-1* positive (Table 1). On fetal US, the congenital hemangioma appears as a well-defined solid mass in which there is an abundance of high-flow vessels and often some internal calcifications. Fetal MRI is helpful in defining the extension of the congenital hemangiomas, especially important in facial (intracranial extension) and trunk types (intraspinal extension) and to predict neurological symptoms. This helps in distinguishing congenital hemangioma from mimickers having a similar appearance with high-flow mass, such as arteriovenous malformation, kaposiform hemangioendothelioma, sacrococcygeal teratoma, rhabdomyosarcoma, sarcoma, and neuroblastoma. A congenital hemangioma, similar to an arteriovenous malformation, presents hyperplastic afferent and efferent vessels with shunting and potential cardiac overload, but, unlike arteriovenous malformation which consists of only vessels, it is a solid tumor. Although rare, teratoma and (rhabdomyo)sarcoma can be mixed, solid and cystic, hypervascular, and congenital forms, and may mimic congenital hemangioma (Fig. 6). Multiple hepatic hemangiomas can have a similar multifocal appearance as congenital metastatic neuroblastoma; however, they can be differentiated by the restriction of diffusion, as well as the presence of an adrenal or paravertebral tumor (Fig. 5).

#### Kaposiform hemangioendothelioma

Kaposiform hemangioendothelioma is a congenital high-flow benign vascular tumor with an aggressive behavior, as it grows invasively through fascial planes. On fetal imaging, the hypervascularity and invasive growth can be diagnostic. At birth, it can be present as a warm, red-purple, and painful swelling that may be confused with a congenital hemangioma, venous malformation, or even birth-related trauma or infection. However, kaposiform hemangioendothelioma continues to grow after birth and is associated with severe thrombocytopenia, hemolytic anemia, and consumptive coagulopathy, a potentially life-threatening condition known as Kasabach-Merritt syndrome [8]. Therefore, biopsy of a congenital high-flow and continuously growing mass is usually advised, for microscopic and genetic evaluation, enabling prompt, optimal, and curative treatment [9].

## Vascular malformations of the fetal central nervous system

Vascular malformations of the fetal central nervous system represent a rare subgroup of anomalies caused by abnormal development of the lymphovascular system in the brain and spinal cord. They are difficult to diagnose antenatally due to their rarity and overlapping imaging features, but also as they may be insignificant during fetal life and only become symptomatic postnatally. Of these, dural sinus arteriovenous malformations and vein of Galen malformations may be detected antenatally.

### Dural sinus arteriovenous malformations

**Fig. 7:**
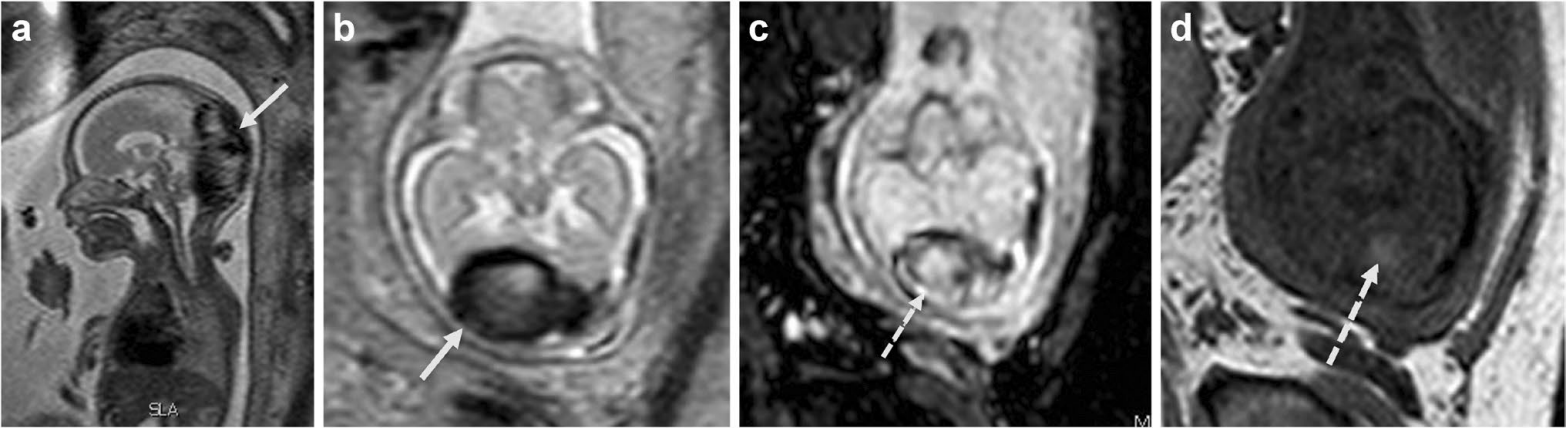
Dural arteriovenous malformation in a male fetus at 25 weeks gestational age whos had ventriculomegaly and an anechoic midline structure of the posterior fossa on prenatal ultrasound (not shown), for which magnetic resonance imaging was indicated. **a**, **b** Sagittal (**a**) and axial (**b**) T2-weighted images reveal a prominent extra-axial, heterogenous flow-void (*arrow*) at the posterior part of the cranium, in keeping with dural arteriovenous malformation. **c**, **d** On axial T2* (**c**) and T1-weighted (**d**) images, a round structure (*broken arrow*) is identified within the flow-void, suggesting a thrombus

Dural arteriovenous malformations are uncommon congenital vascular anomalies, of unknown etiology, probably related to the persistence of physiological ballooning of the fetal dural sinuses [10, 11]. Although they are not the usual suspects, when identified antenatally, they present as anechoic structures along the anatomic sites of venous sinuses, most commonly in the posterior fossa. They cause massive sinus dilatation, in the background of arteriovenous slow-flow shunts between the dural sinus and choroidal arteries and they are invariably complicated by thrombosis of the involved sinus [10, 11]. On US, the dilated sinus may appear as a cystic, slightly hyperechoic structure, whereas Doppler examination does not always add value as slow flow may not be identified. Denser hyperechoic areas implying development of thrombosis within the dilated sinus, associated ventriculomegaly, craniomegaly, and hydrops, may be encountered requiring the performance of fetal cranial MRI. The latter enables confirmation of the presence of an expansile, heterogenous, medium intensity lesion at the site of the affected dural sinus, more often in the posterior part of the skull, causing compression of the adjacent cerebellum. Within this lesion, round areas of hyperintensity on T1-weighted images and hemosiderin deposits on susceptibility sequences are usually identified in keeping with the presence of a thrombus [10, 11]. Depending on the size of the dilated sinus, obstructive hydrocephalus may develop causing increased biparietal and fronto-occipital diameters (Fig. 7) [10]. Fetal MRI allows for the assessment of the brain parenchyma and rules out the presence of associated parenchymal ischemic or hemorrhagic lesions with additional use of diffusion sequence. Overall, these malformations may cause heart failure and are then associated with an uncertain outcome. They may however regress spontaneously, even if substantial in size. Therefore, close monitoring and imaging of the fetus is advised. On imaging, the presence of parenchymal brain lesions, severe ventriculomegaly, arteriovenous shunts, and hydrops is indicative of poor outcome, whereas regression of the malformation and/or increase of the size of thrombus with absence of signs of heart failure are associated with a good prognosis [10].

### Vein of Galen malformation

**Fig. 8:**
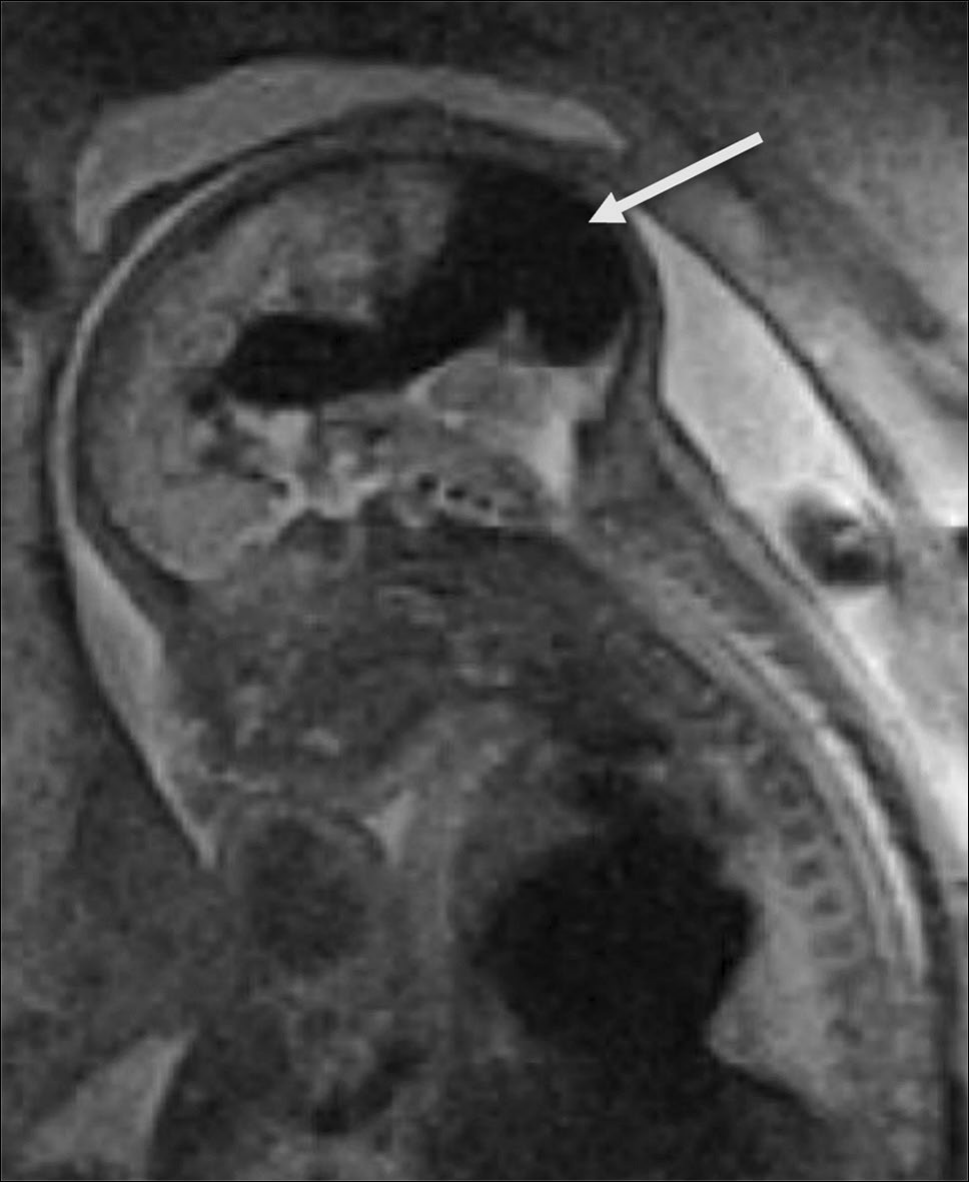
Vein of Galen malformation. Fetal magnetic resonance imaging in a 23-week gestational age male fetus with a vascular, midline posterior supratentorial structure that showed turbulent flow on color Doppler ultrasound (not shown). Sagittal T2-weighted image confirms the presence of an aneurysmal flow void along the course of internal cerebral veins and, more prominently, of the straight sinus (*arrow*) in keeping with Vein of Galen malformation

Vein of Galen malformation is a rare congenital malformation of the cerebral veins, accounting for 1% of fetal intracranial malformations [12]. It arises early in gestation due to the development of a fistula between cerebral arteries and the deep prosencephalic vein, a precursor of the vein of Galen. Eventually, during late gestation, it becomes a significantly dilated, accumulating aneurysmal configuration [12]. This process often causes injury to the brain parenchyma and congestive cardiac failure, both associated with poor prognosis. The imaging findings, which are usually revealed late in gestation, are specific: US reveals an anechoic tortuous midline structure, posteriorly to the third ventricle, with typical color Doppler features of turbulent arterial and venous flow. Fetal MRI, recommended for these fetuses, confirms the presence of the midline, aneurysmal, and tortuous, vascular midline structure with signal void. Importantly, fetal MRI scrutinizes the relationship of the mass and its effect to adjacent parenchyma, focusing on areas of parenchymal ischemia and white matter volume loss (Fig. 8) [12]. Prognosis in these fetuses is affected by the degree of cardiac failure, related to the number of arteriovenous shunts, which is awkwardly higher in the smaller-sized lesions that cause higher degree of straight sinus dilatation. Conversely, increased venous pressure in the brain parenchyma caused by large-sized malformations is also associated with ischemic changes and poor prognosis [12, 13].

### Intraspinal arteriovenous malformations

It is extremely rare to identify intraspinal arteriovenous malformations antenatally due to the small size of the fetal spinal canal and cord. In the presence of extensive subcutaneous arteriovenous malformations or large vascular tumors, fetal MRI may suggest intraspinal extension and identify the level of affected cord, thus aiding parental consultation, delivery planning, and perinatal management.

## Glymphatics

Recent new insights reveal that the lymphatic function of the brain parenchyma and myelum is facilitated by the cerebrospinal fluid (CSF) and perivascular and peri-glial fluid spaces; these form the glymphatic system. Congenital and developmental abnormalities in children have been associated with enlarged CSF spaces, such as hydrocephalus, as well as autism spectrum disorders and schizophrenia [14]. However, the precise mechanism needs to be further investigated; according to the current knowledge, congenital abnormalities in the glymphatics are not yet considered to be in the spectrum of intracranial lymphatic malformations.

## Syndromic and complex vascular malformations

If a mutation affects the *PIK3CA* gene, which is responsible for cell proliferation and angiogenesis, an overgrowth of the affected tissues will result in abnormal architecture and circulation of various phenotypes included in *PIK3CA*-related overgrowth syndrome or spectrum (PROS). Two of these rare syndromes may be identified antenatally, the CLOVES (congenital lipomatous overgrowth with vascular malformations, epidermal nevi, and skeletal anomalies/scoliosis) syndrome and the Klippel-Trenaunay syndrome (Table 1). In CLOVES syndrome, variable vascular malformations, and even spinal arteriovenous malformations, may be identified together with excessive lipomatous tissue or features of hemimegalencephaly and gyration anomalies (macrocephaly-capillary malformation, M-CM) [15]. In Klippel-Trenaunay syndrome, slow-flow vascular malformations may present in combination with an overgrowth of bone and soft-tissue (Fig. 3).

Sturge-Weber syndrome presents with facial capillary port-wine stain lesions along the territories of the ophthalmic (V1) and maxillary (V2) branches of the trigeminal nerve (V), as well as leptomeningeal microvenular dysplasia, which impairs brain perfusion due to blood stasis [9]. According to reports, the latter may cause cortical malformation, such as polymicrogyria and ipsilateral cerebral atrophy, gliosis, and calcifications, evident not only during early childhood but also in the prenatal imaging, with typically reduced signal of the affected, atrophic hemisphere on T2-weighted images [16].

## Conclusion

The lymphatic, venous, and arteriovenous malformations together with the vascular tumors that may be discovered on prenatal US and fetal MRI, as well as their mimickers, are presented in this overview. Most of the lesions are benign and may even involute spontaneously. However, there are certain vascular anomalies that require detailed diagnosis and specific supportive and therapeutic care. Low-flow lymphatic malformations in the neck may need perinatal care to secure the patency of the airways. High-flow anomalies may need postnatal imaging in monitoring the presence of high-output cardiac failure and possibly a biopsy, to distinguish them from malignancies and to decide on appropriate therapeutic procedures.

## Data Availability

Not applicable
